# Online Platform as a Tool to Support Postgraduate Training in General Practice – A Case Report

**DOI:** 10.3205/zma001136

**Published:** 2017-11-15

**Authors:** Lorena Dini, Claire Galanski, Susanne Döpfmer, Sabine Gehrke-Beck, Gudrun Bayer, Martin Boeckle, Isabel Micheel, Jasminko Novak, Christoph Heintze

**Affiliations:** 1 Charité – Universitätsmedizin Berlin, Institute of General Practice, Berlin, Germany; 2 EUROPEAN INSTITUTE FOR PARTICIPATORY MEDIA, Berlin, Germany

**Keywords:** Postgraduate training, general practice, online platform, case-based learning, social web, Physician Competency Framework

## Abstract

**Objective:** Physicians in postgraduate training (PPT) in General Practice (GP) typically have very little interaction with their peers, as there is usually only one resident physician working in their respective department or GP office at a given time. Therefore, the online platform KOLEGEA, presented here, aims to support postgraduate training in general practice (PT in GP) in Germany through virtual interaction.

**Methodology:** In 2012, the interdisciplinary research project KOLEGEA set up an online platform that any physicians in PT in GP can use for free after registration with their unitary continuous education number (Einheitliche Fortbildungsnummer, EFN). It offers problem-based learning and allows to discuss self-published anonymized patient cases with the community that can be classified and discussed with experienced mentors (specialists in general practice - GPs) in small virtual groups.

**Results:** An anonymous online survey carried out as part of the 2014 project evaluation showed a good acceptance of the platform, even though shortage of time was mentioned as a limiting factor for its use. Data analysis showed that KOLEGEA was used by PPT in GP in all federal states. Patterns of passive use were predominant (90%). This report also describes the further development of the platform (in 2015 and 2016) that integrates an activity monitor as part of a gamification concept.

**Conclusions:** Due to a low response rate of the 2014 online survey and the preliminary evaluations of usage patterns we could identify only initial trends regarding the role of KOLEGEA in supporting PPT. The platform was perceived as a helpful supplement to better structure PT in GP.

## Zusammenfassung

**Zielsetzung: **Ärztinnen und Ärzte in Weiterbildung (ÄiW) Allgemeinmedizin haben überwiegend wenig Austausch mit ihrer Peer Group, weil sie meist die einzigen ÄiW sind, die zu einer gegebenen Zeit in dem entsprechenden Fachbereich bzw. der Praxis arbeiten. Die hier vorgestellte Online-Plattform „KOLEGEA“ hat daher das Ziel, die Weiterbildung Allgemeinmedizin in Deutschland durch den virtuellen Austausch zu unterstützen.

**Methodik: **In einem interdisziplinären Forschungsprojekt wurde 2012 eine Online-Plattform etabliert, die Ärztinnen und Ärzten in Weiterbildung (ÄiW) mittels ihrer Einheitlichen Fortbildungsnummer (EFN) frei nutzen können. Neben problemorientierten Lernen können anonymisiert eingestellte Patientenfälle mit der Gemeinschaft diskutiert und mit erfahrenen Mentoren (Fachärztinnen/Fachärzten für Allgemeinmedizin) in virtuellen Kleingruppen eingeordnet und diskutiert werden.

**Ergebnisse:** Eine anonyme Online-Umfrage im Rahmen der Evaluation in 2014 wies darauf hin, dass die Plattform positiv aufgenommen wurde, wenn auch Zeitmangel als begrenzender Faktor für die Nutzung beschrieben wurde. Die Datenanalyse zeigte, dass die KOLEGEA-Plattform in allen Bundesländern von ÄiW genutzt wurde. Passive Nutzungsmuster überwiegen (90%). Ergänzend wird die Weiterentwicklung der Plattform in 2015/2016 beschrieben, die einen Aktivitätsmonitor als spielerische Lernelemente integriert.

**Schlussfolgerungen: **Die niedrige Response der Online-Umfrage 2014 und die vorläufigen Auswertungen zu Nutzungsmustern lassen nur erste Trends erkennen, ob KOLEGEA den Weiterbildungsprozess unterstützt. Die Plattform wird als hilfreiche Ergänzung zu einer besseren Strukturierung der Weiterbildung Allgemeinmedizin eingeschätzt.

## 1. Introduction

A wide range of initiatives aiming to increase the number of young professionals choosing the GP specialization are currently under development, as a response to the growing shortage of GPs, especially in rural regions. Support measures for general practice are currently being initiated in Health Policy [1] through bill for the provision of healthcare service (Versorgungssträkungsgesetz). In the future, PT in GP is likely to be funded through closer cooperation between the association of statutory health insurance physicians (Kassenärztliche Vereinigungen), the regional medical chambers (Landesärztekammern), The German Hospital Federation (Deutsche Krankenhausgesellschaft) and the university-based institutes of GP education [2].

PPT in GP usually have very little interaction with their peers since there is often only one physician in PT working at a given time in their respective department or GPs office.

Modern forms of communication can improve structural conditions and offer new opportunities for further education and training. The internet in general and network-based learning in particular play an important role in making new knowledge available to both educators and learners [3]. Web 2.0 has already started shaping GP undergraduate medical studies. Looking towards the future, it is important to make more explicit use of the unique possibilities of the internet, not only for medical teaching [4], but also for PT.

This article presents an innovative project that supports PT in GP by the means of an online platform. Virtual knowledge exchange [5], [6] is seen as a promising complementary addition to existing training offers that foster PT. Young physicians, are familiar with the didactic approach of problem-oriented and self-directed learning by means of clinical case studies, which KOLEGEA is modeled on. Following this well-established learning approach [7], the online platform for "Cooperative Learning and Mobile Communities for Continuing Professional Education in General Practice" KOLEGEA (“Kooperatives Lernen und mobile Gemeinschaften für berufsbegleitende Weiterbildung Allgemeinmedizin“) was developed and tested [8].

## 2. Basis of the KOLEGEA platform

The first step was to design a media-didactic concept based on a quantitative survey of 73 physicians in PT and a focus group. Taking into account the needs of the target group, the KOLEGEA concept was deployed as an online platform. A pilot version was used for three months by a small closed group of users (20 PPT and 1 mentor) and was evaluated at the end of the period [8]. The platform was optimized in terms of design, structure and technology based on the experience of the initial users and the findings of the evaluation. KOLEGEA has been online at https://beta.kolegea.org/kolegea since September 2013 and can be used nationwide for free by PPT or licensed physicians [5]. Users can register with their EFN-continuous education number regardless of where they are based. The platform is the first German online portal that allows for a virtual exchange of clinical-case-based knowledge. Registered members can anonymize patient cases they encountered in their doctor's office and share these with the KOLEGEA community after having formulated a specific question in regards to the case. This allows physicians to discuss their current patients with experienced GPs and other PPT, to gain new insights, perspectives to find new solutions or learn approaches from each other.

**Figure 1:**
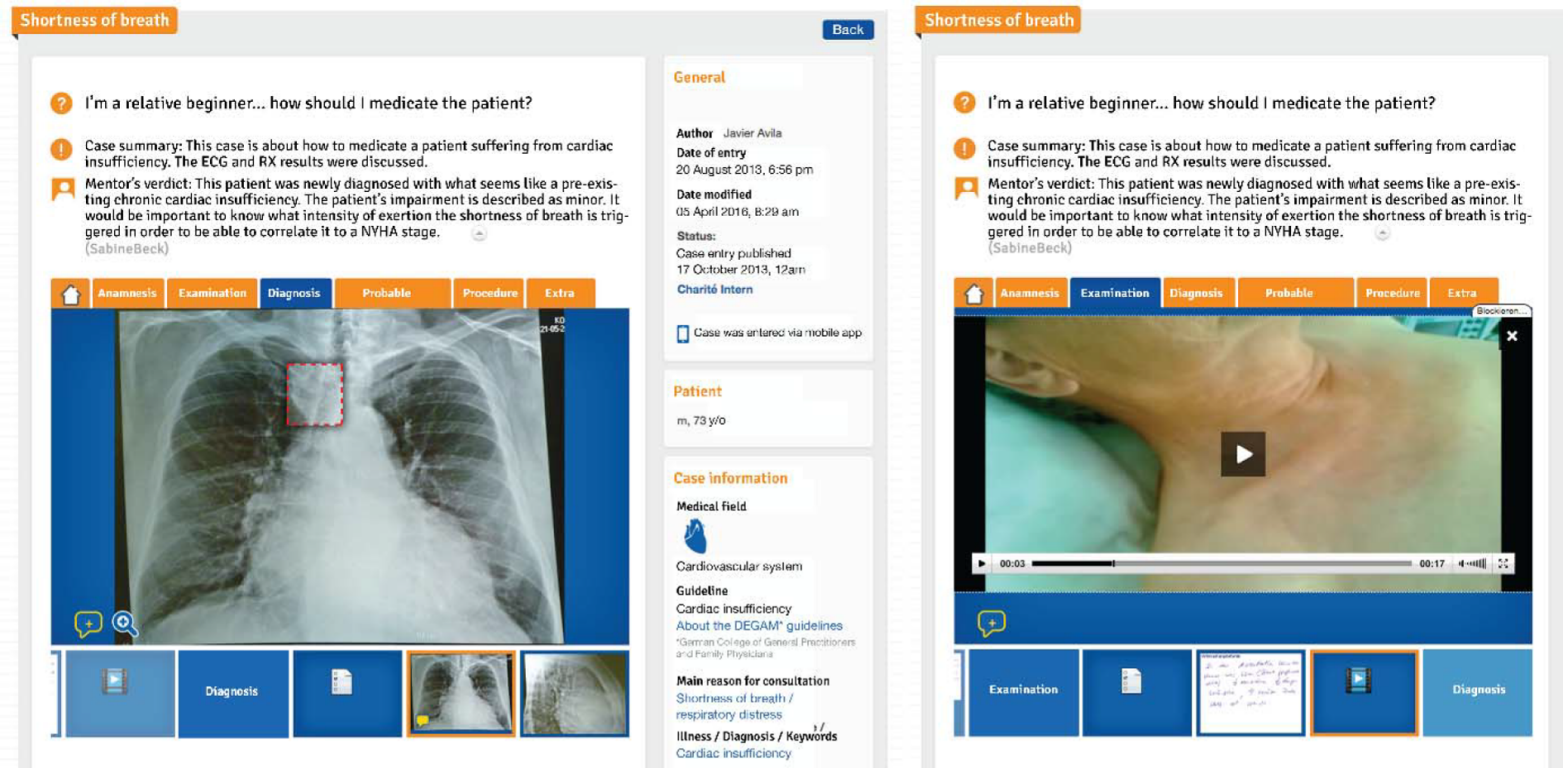
Example for a case entry

The structure of the case presentations on KOLEGEA is based on the usual structure of clinical practice (anamnesis, examination, diagnostic testing, differential diagnosis, procedure). Multimedia data such as photos, ECG, X-rays, videos or sound recordings can be added to the clinical case description. The case entries can also be linked to relevant clinical guidelines, journal articles or helpful internet links (see Figure 1 (Fig. 1)). Initially, 20 structured patient cases were published on the platform by experienced GPs in order to motivate PPT to publish their own clinical cases.

Experienced GPs support PPT by being temporarily active as “mentors” on the platform. Initial needs assessment showed that PPT consider support by GPs mentoring a helpful training supplement. Mentors comment on the described cause for consultation, on possible differential diagnosis and provide the perspective on the clinical approach from a specialist point of view.

To stimulate engagement and boost the number of activities on the platform, the users receive an automatic weekly notification e-mail with a list of the latest activities conducted on the platform.

## 3. Evaluation of the original platform: online user survey

In an anonymous online survey in June 2014, all registered users (n=198) were asked about their platform usage behavior, as well as their perceived strengths and limitations of the application. The survey collected quantitative and qualitative user feedback on relevant quality features using 5-stage Likert scales and open questions, e.g. perceived usefulness, ease of use, satisfaction and enjoyment of use. Furthermore, the survey also collected feedback about usage behavior and experience, as well as ideas regarding future application possibilities for the platform. Approximately 10% of registered users (17 participants) completed the evaluation [8].

Descriptive results showed that users who took part in the survey used the internet for approximately two hours a day (SD=1.74, range 0.5–7.0) and logged into the platform on average five times per month (SD=7.22, range 1–30). The majority of respondents (88.2%) mainly used KOLEGEA during their leisure time. The main limiting factor for use was "professional life leaves too little time for self-study", followed by "lack of participation of other participants" and "technical difficulties in use". For half of the respondents, the strongest motivation to engage on the platform was the weekly notification email. Reasons for passive behavior (i.e. not engaging in discussions of cases) were: “lack of time”, "cumbersomeness of use" and being a "new platform member" as well as "low involvement of the other users". The functions for creating and discussing cases were perceived as particularly important. 75% of participants also found mentoring by experienced GPs to be helpful. 88.3% considered the KOLEGEA platform to be a "useful supplement to existing e-learning courses available online" [8].

Users particularly appreciated the opportunity for professional exchange and relevance of the presented patient cases for PT. Furthermore, the target group appreciated the possibility to be anonymous. Suggestions for change and/or improvement were related to the design (clarity of structure of the platform and categorization of cases) and more engagement from other users, including peers and mentors. It is important to note that the survey response rate was low, and that therefore only limited statements can be made regarding usage patterns on the platform.

## 4. Evaluation of the original platform: anonymous database analysis

In December 2015 an anonymous database analysis of KOLEGEA platform user behavior was conducted. At that time, there were 298 registered users from 16 different countries. This corresponds to the usual degree of dissemination of new online platforms in the initial establishing phase [9]. Almost half of the users chose an "anonymous" username. Only 31% of users indicated their gender. The non-sharing of personal data points to KOLEGEA users’ need for anonymity. The vast majority of users were in the early stage of their careers, with many users doing their PT in Berlin (28%), Bavaria (16%), Baden-Württemberg (11%) and North Rhine-Westphalia (7%) [10].

Over 100 clinical cases were discussed during the designated research period [8], [11]. The vast majority of KOLEGEA users were passive readers who did not actively participate or publish any patient case entries. A few individual users ("lead users") on the other hand were very active, making the majority of new patient cases entries or participating in discussion of other cases. This behavior reflects a known pattern of activity in online communities that describes the ratio of active users (about 10%) to passive users (about 90%) [12].

## 5. Further development of the KOLEGA platform

Based on this evaluation, the platform was further developed with a follow-up financial grant by the German Federal Ministry of Education and Research (Bundesministerium für Bildung und Forschung, BMBF). The new focus was to improve the contents structure according to medical competencies, as well as integrating gamification (game-based features) into the platform.

### 5.1 Physician Competency Framework in General Practice and CanMEDS

The standard medical procedure in patient consultation in GP is based on the patient’s motives of consultation. Special attention was therefore paid to this aspect when further developing the KOLEGEA platform. The Physician Competency Framework General Practice (Kompetenzbasiertes Curriculum Allgemeinmedizin, KCA) [http://www.kompetenzzentrum-allgemeinmedizin.de/public/curriculum.shtml], which is currently used in Germany, formed the basis for the further conceptual development of the platform. The KCA lists 78 reasons to seek consultation corresponding to twelve medical areas. In order to improve the platform, the motives of consultation from already published cases were analyzed and matched with the KCA list. Finally, the list was condensed to a manageable number of 73 main reasons for consultation. 

Table 1 (Tab. 1) shows a side-by-side comparison of the medical areas as defined by the KCA and those previously included in the KOLEGEA platform.

The early inclusion of competency-based training in PT for GP has been receiving more attention in the later years. [13], [14]. The Canadian CanMEDS competency-based framework (last updated in 2014 [15], [16]) was used as an effective guiding concept in the further development of KOLEGEA.

**Figure 2:**
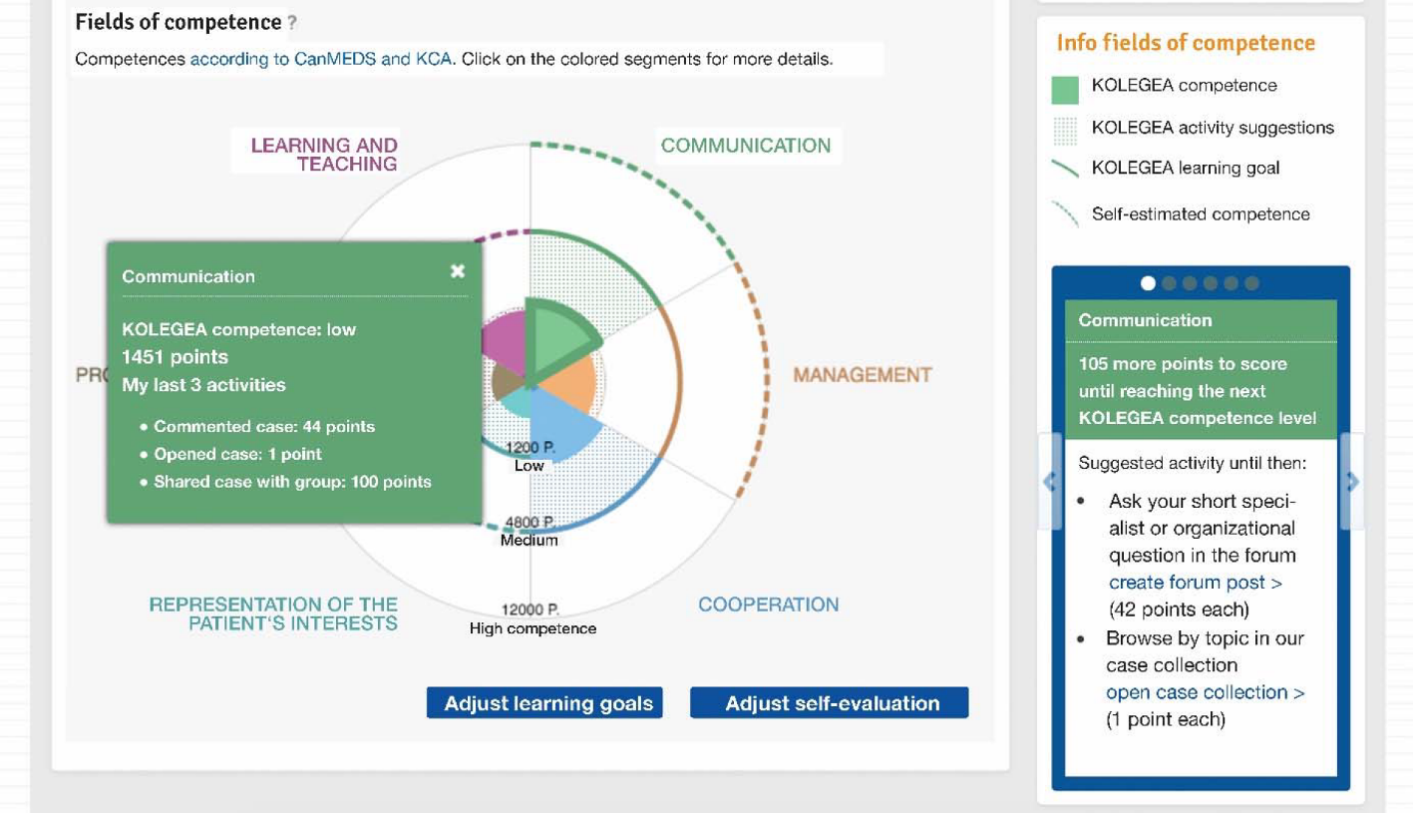
The 6 key competences on KOLEGEA (activity monitor, 1^st^ part)

In the CanMEDS framework, knowledge, skills and abilities are consolidated into six core competencies in addition to the medical expertise and organized thematically around seven key physician roles: medical expert, communicator, collaborator, manager, health advocate, scholar and professional. The six core competencies were also incorporated into the KCA (see Figure 2 (Fig. 2)).

#### 5.2 Integrating gamification

In order to make it more attractive to users and increase the end-user motivation, game-based features (also called gamification elements) were integrated into the further development of the platform. Gamification is the application of game-design elements and game principles in non-game contexts. This includes, for example, principles of rewards and competition [17] introduction of incentives to motivate learners and to promote targeted learning [18], [19].

Observations in medical contexts [20], [21] have shown that this can boost users’ motivation and increase their level of engagement on a given platform. It has also been observed and ascertained that introducing incentive systems effectively boosts the generation and sharing of content [22]. Further, the feeling of belonging to a social group and the perception of a personal benefit is described as a relevant factor [23], [24].

There are two main types of gamification:

1. using different forms of point systems and reputation systems (points, medals, badges, status levels, etc.) that place the focus on personal rewards or comparisons with other users.
2. developing so-called “serious games” that integrate solving pragmatic tasks, knowledge acquisition or that embed learning into a game specially designed for this purpose.

For KOLEGEA, a combination of the two incentive models was developed and integrated into the platform. 

#### 5.3 Gamification and CanMEDS competencies

**Figure 3:**
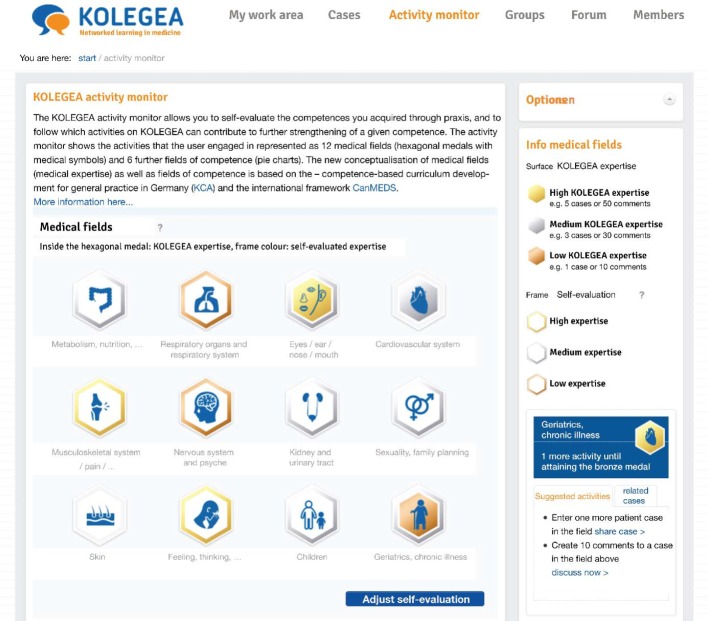
12 Medical areas at KOLEGEA (activity monitor, 2^nd^ part)

On the platform, gamification is combined with CanMEDS competencies, which are central to many international PT in GP programs. Each user can see the activities they performed on the platform on their personal "activity monitor", which visualizes the online activity on the platform as an online learning progress relating to the development of each area of medical expertise and the CanMEDS competencies. On the KOLEGEA platform this learning progress in the user’s level of expertise is visualized by the color coding (see Figure 3 (Fig. 3)). The activity monitor is a tool for users to individually adjust and angle their activity on KOLEGEA to best suit their further PT learning objectives. It must be noted that the user’s self-assessment and the activities conducted online represent only a simple estimate of the user’s expertise on a particular competence, and this has to be distinguished from actual knowledge and skills in real practice (see Figure 2 (Fig. 2)).

Case-based activity (i.e. sharing a patient case from one’s practice and participating in the discussion about a case) is visualized as different hexagonal “medals” bearing medical symbols and represent the twelve medical areas (see Figure 3 (Fig. 3)). For this purpose, the Institute of General Practice of the Charité (Institut für Allgemeinmedizin der Charité) slightly adapted the twelve medical areas of the KCA.

The different colors of the inner surface of the medal (three levels: bronze, silver, and gold) automatically show the level of activity corresponding to the medical expertise area that each user has achieved on the platform. The golden medal indicates a very high KOLEGEA activity in a specific medical area. The progress in the Expertise for a given medical area is visualized in three levels: low, medium, and high with a different medal color corresponding to each level. Based on the individual activity profile, KOLEGEA makes suggestions promoting specific activities (e.g., "Share another case in the medical field ‘geriatrics, chronic illness’").

## 6. Discussion

The low response rate of the 2014 online survey and the preliminary evaluations of usage patterns reveal only first trends as to whether KOLEGEA benefits the PT process. The platform was considered a helpful supplement to better structure PT in GP. International studies have shown that GPs are generally interested in e-learning services and appreciate the flexible time planning that online-learning offers, as well as the possibility of being able to build on their own self-motivation. In Anglo-Saxon countries in particular, e-learning programs are already popular with GPs [25]. KOLEGEA was successfully established as the first online platform for Germany that is specifically tailored to the needs of PPT in GP [26].

KOLEGEA’s pedagogical approach and additional benefit is based on supporting online interaction among scholars in a learning community as a “community of practice” [27]. Additionally, the KOLEGEA approach is based on building a collection of learning objects, such as the patient cases and discussions about these, generated and provided by the learners themselves [28], [29].

In addition to contributing to the deepening of users’ medical expertise, the platform’s structure can also support strengthening the core GP competencies [30], [31], [32]. Promoting the competency-based model will also become more important for GPs in academia and mentors and GPs in charge of PPT [33].

An important aspect is the chosen anonymity of most KOLEGEA users on the platform to remain unidentifiable by other users (PPT and GPs). A further development could include the regionalization of virtual groups, allowing users to engage in direct interaction in regional groups alongside the anonymous online communication with the whole community. With regards to existing PPT training networks (Weiterbildungsverbünde) as well as the imminent establishment of competence centers new potential for usage emerge. Interaction in closed-groups with regional or local mentors within a specific competence-center or Institute of General Practice could be an interesting additional supplement.

“Recommendation” functions are a suitable instrument to support the efficient exploration of large data sets [34]. Content-based processes analysis detect similarities between text vectors or available keywords. The quality of the calculated recommendations primarily depends on the quality of the database used [35]. KOLEGEA provides “recommendation” functions to all users. At present, however, it remains unclear whether the quality of the current database is high enough for these recommendations to be of a considerable benefit to PPT.

Gamification has been successfully used as an effective incentive mechanism for the information and knowledge exchange in other fields [17], [21], [36], but has not yet been applied in GP. While sparse, previous research shows encouraging effect for the use of gamification in PPT [37]. However, in light of the pronounced time constraints of young doctors, it is challenging to develop an incentive system specifically tailored to this target group.

## 7. Outlook

Clinical case-based online communication and knowledge sharing using a tool that allows self-assessment of competencies is perceived to be a valuable support of PT in GP through web 2.0 applications. It is necessary to examine the extent to which the virtual networking of PPT should be strengthened by means of getting personally acquainted, or with accompanying seminars, as an additional measure. KOLEGEA could be an apt helpful addition to the emerging GP competence centers.

Future evaluations should examine whether the dissemination and long-term use of the platform can contribute to improving the quality, effectiveness and attractiveness of PT in GP.

## Acknowledgement

Research groups “Interaktive Systeme und Kooperative” (Prof. J. Ziegler), “Lernunterstützende Systeme” (Prof. H-U. Hoppe) of the University of Duisburg-Essen and TheCode AG. 

## Funding

This project was co-funded by the Federal Ministry of Education and Research (Bundesministerium für Bildung und Forschung, BMBF) and the EU’s European Social Fund (funding number 898 14145).

## Competing interests

The authors declare that they have no competing interests. 

## Figures and Tables

**Table 1:**
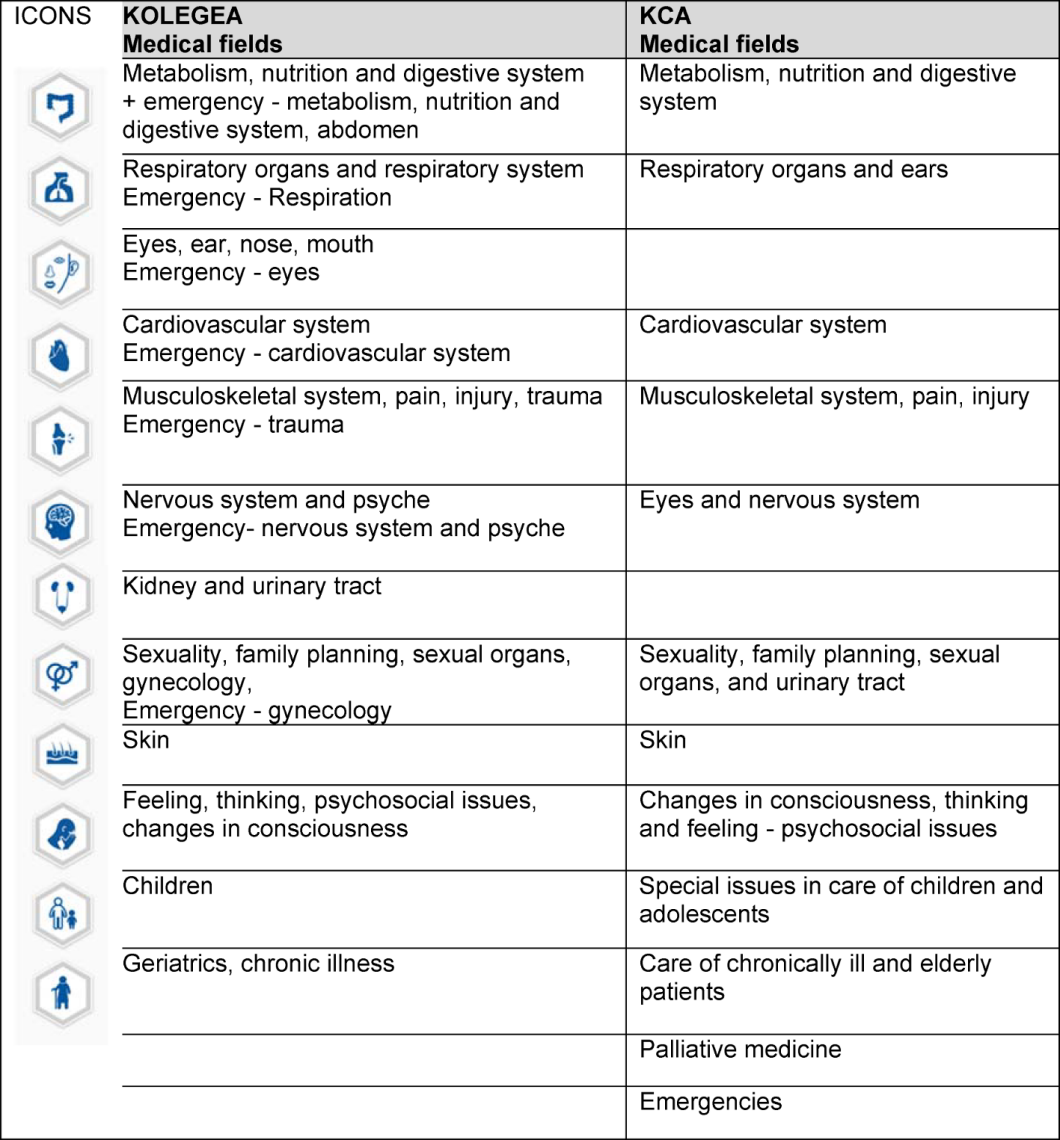
Comparison of 12 medical fields: KOLEGEA and KCA
